# Dose–Response Relationship Between BRAF V600E Abundance and Cervical Lymph Node Metastasis in Papillary Thyroid Cancer

**DOI:** 10.3390/cancers17213562

**Published:** 2025-11-03

**Authors:** Yisikandaer Yalikun, Yuxin Shen, Anyun Mao, Qianlei Zhou, Jinchen Wei, Yue Zhu, Miaoyun Long

**Affiliations:** 1 Department of Thyroid Surgery, Sun Yat-sen Memorial Hospital, Sun Yat-sen University, Guangzhou 510030, China; yalikun@mail2.sysu.edu.cn (Y.Y.); shenyx53@mail2.sysu.edu.cn (Y.S.); 2 Department of Physician Services, Xinjiang International Medical Center (Xinjiang International Hospital), Urumqi 830017, China; 3 Department of Thyroid and Breast Surgery, Dongguan Houjie Hospital, Dongguan 523000, China; maoanyun@163.com; 4 Department of General Surgery (Thyroid Surgery), The Sixth Affiliated Hospital, Sun Yat-sen University, Guangzhou 510655, China; zhouqlei5@mail.sysu.edu.cn (Q.Z.); weijch6@mail2.sysu.edu.cn (J.W.); 5 Biomedical Innovation Center, The Sixth Affiliated Hospital, Sun Yat-sen University, Guangzhou 510655, China

**Keywords:** papillary thyroid carcinoma, BRAF V600E, mutation abundance, machine learning

## Abstract

**Objectives**: Papillary thyroid carcinoma (PTC) frequently presents with cervical lymph node metastasis (CLNM), yet preoperative tools often encode BRAF V600E as a binary variable, potentially overlooking information contained in mutation abundance. We sought to quantify the dose–response relationship between BRAF V600E abundance and CLNM and to develop an interpretable model for preoperative risk stratification. **Methods**: We performed a single-center retrospective study of consecutive PTC patients who underwent preoperative BRAF V600E testing and surgery from 2019 to 2023. Patients were randomly split 70/30 into training and test sets. Candidate predictors included clinical and ultrasound features and BRAF V600E abundance. We used multivariable logistic regression and restricted cubic splines (RCS) to assess nonlinearity and compared six machine-learning algorithms (LR, KNN, SVM, XGB, LightGBM, and NN). Model performance was evaluated by F1, AUC, calibration, and decision-curve analyses; SHAP aided interpretation. Ethics approval: SYSKY-2024-169-01. **Results**: The cohort included 667 patients; CLNM occurred in 391 (58.6%). CLNM cases had higher BRAF abundance (median 23% vs. 17%) and characteristic clinical/sonographic differences. RCS revealed a nonlinear association between abundance and CLNM, with a steep risk rise of up to ~20.7% followed by a plateau. Among six algorithms, XGBoost showed the best validation performance (AUC 0.752; F1 0.73). SHAP indicated that maximum tumor diameter, BRAF abundance, age, and microcalcifications contributed most to predictions. **Conclusions**: Modeling BRAF V600E as a quantitative abundance—rather than a binary status—improves preoperative CLNM risk assessment in PTC. An interpretable XGBoost model integrating abundance with routine features demonstrates acceptable discrimination and potential clinical utility for individualized surgical planning and counseling.

## Simple Summary

Cervical lymph node status is critical when planning surgery for papillary thyroid carcinoma, yet most tools treat the BRAF V600E mutation as a simple yes/no finding. We asked whether the amount of BRAF (reported as a percentage in fine-needle aspiration samples) provides more useful, patient-specific information. In a single-center cohort, we measured BRAF abundance preoperatively and linked it with surgical pathology, using flexible models that allow for a relationship where risk increases as abundance rises rather than a fixed cut-off. We found a steep rise in nodal risk at around 20.7% BRAF abundance, followed by a near-plateau, and built an easy-to-understand risk tool that combines BRAF with routine ultrasound features and demographics. This approach may help clinicians discuss options before surgery, focus attention on higher-risk patients, and avoid unnecessary procedures in lower-risk cases. Prospective, multi-center validation is still needed.

## 1. Introduction

Thyroid cancer is the most common malignant tumor of the endocrine system, among which papillary thyroid carcinoma (PTC) accounts for more than 80% [1,2]. Although PTC generally has a favorable prognosis (approximately 90% 10-year overall survival), occult cervical lymph node metastasis (CLNM) or distant metastasis can still lead to adverse outcomes [3,4]. As a key prognostic factor, CLNM significantly increases the risk of recurrence and reduces treatment efficacy [5,6,7,8], and its occurrence is closely associated with patient prognosis [9]. Accordingly, timely and accurate prediction of CLNM risk is crucial for optimizing individualized treatment and improving outcomes [10,11]. Against this background, developing an effective and readily interpretable early-identification model to target populations at high risk for CLNM has become one of the core objectives for improving long-term efficacy.

In recent years, progress has been made in predicting CLNM risk in PTC; however, models based on ultrasonography and conventional clinical parameters remain limited: they often rely on single predictors, offer insufficient discriminative accuracy and clinical decision support, and fail to meet the demands of precise preoperative evaluation [12,13,14]. Machine learning (ML), capable of integrating multidimensional data, has shown value in disease prediction—particularly in risk assessment for PTC [15,16,17]—and multiple models have been constructed based on clinical and imaging features [18,19]. Nevertheless, many studies have not effectively incorporated genomic data, or have encoded gene mutations simply as binary variables (present/absent), overlooking the potential information contained in mutation abundance, which has resulted in suboptimal performance and inconsistent findings [20,21,22].

Among these genetic alterations, BRAF V600E is one of the most common mutations in PTC, carried by approximately 45–60% of patients and closely associated with tumor initiation, progression, and adverse prognosis [23,24,25]. At the same time, some studies have not identified a significant association with prognosis; such work often remains at the level of mutation presence/absence without further examination of mutation abundance, limiting understanding of tumor heterogeneity and mutational burden [26,27,28]. As an important indicator for quantifying heterogeneity and mutational burden, mutation abundance is expected to provide a more refined delineation of risk; however, existing explorations are often constrained by small sample sizes, limited evaluative dimensions, and a lack of interpretable predictive models [29,30,31,32].

This study aims to integrate BRAF V600E mutation abundance with clinical characteristics to evaluate its clinical value in PTC: (i) to elucidate the dose–response relationship between BRAF V600E mutation abundance and CLNM and to propose a clinically actionable risk threshold; and (ii) to develop and validate an interpretable ML predictive tool that combines BRAF mutation abundance with clinical variables, thereby improving the accuracy and operability of preoperative CLNM assessment in PTC and providing theoretical rationale and practical guidance for individualized treatment planning.

## 2. Materials and Methods

### 2.1. Data Sources

This study was approved by the Ethics Committee of Sun Yat-sen Memorial Hospital and conducted in accordance with the Declaration of Helsinki. Consecutive patients hospitalized in the Department of Thyroid Surgery between 1 September 2019 and 1 September 2023 who underwent preoperative fine-needle aspiration (FNA) with BRAF testing and were postoperatively confirmed as papillary thyroid carcinoma (PTC) were screened.

The primary outcome was any cervical lymph node metastasis (CLNM), encompassing the central (level VI) and/or lateral compartments (levels II–V). Pathology served as the reference standard for compartments that underwent dissection (routine CND; selective LND when indicated).

**Figure 1:**
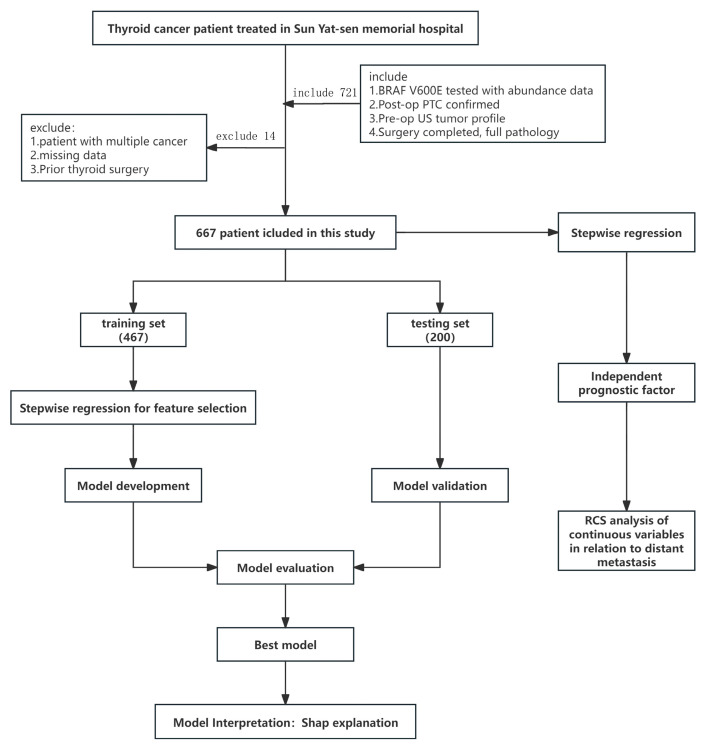
Study design flow chart.

Inclusion criteria: (i) preoperative BRAF V600E testing with available mutation abundance; (ii) postoperative paraffin pathology confirming PTC; (iii) preoperative ultrasound (US) evaluation of tumor characteristics; (iv) definitive surgery with complete postoperative pathology. Exclusion criteria: (i) concomitant malignancies; (ii) incomplete preoperative imaging/genetic data or incomplete pathology; (iii) prior thyroid surgery. All data were de-identified; the requirement for informed consent was waived due to the retrospective use of anonymized data. To ensure data completeness, cases with incomplete records were excluded at pre-enrollment screening; a variable-by-variable audit of the final analytic cohort confirmed 0% missingness for the outcome and all candidate predictors. Accordingly, no imputation or complete-case/sensitivity analyses were performed, and all analyses were conducted on the complete dataset. The overall study workflow is shown in Figure 1.

### 2.2. Clinical Characteristics and Data Processing

Preoperative predictors and outcome. Preoperative variables included demographics; US features (margins, hypoechogenicity, microcalcifications, aspect ratio, CDFI grade, US-suspected cervical lymph node enlargement, US-defined multifocality, US-suspected capsular invasion/ETE, TI-RADS); maximum tumor diameter (cm); and BRAF V600E mutation abundance (%) from FNA. CLNM was defined as metastasis in the central (VI) and/or lateral (II–V) compartments, with postoperative pathology as the reference standard for compartments dissected (CND for all; LND selectively).

### 2.3. NGS

Ultrasound-guided fine-needle aspiration (FNA) was performed on the index (most suspicious) nodule, with two passes per nodule, deliberately sampling distinct regions (quadrants when feasible) to mitigate intratumoral heterogeneity. In multifocal (including bilateral) disease, one of the most suspicious nodules per side was sampled as the index lesion, and other foci were not routinely aspirated. Part of the aspirate was submitted for routine cytopathology and the remainder was immediately transferred to a DNA stabilization tube. Genomic DNA was extracted and quantified on a Qubit fluorometer. Library preparation (fragmentation, end-repair, and adapter ligation) was carried out according to standard procedures, and sequencing was performed on an Illumina MiniSeq platform. Targeted next-generation sequencing was restricted to the BRAF locus (V600E hotspot), using a single-gene capture design optimized for cytology specimens. A minimum on-target depth of ≥500× was required to ensure analytical sensitivity for variant detection. Bioinformatic processing included quality control, alignment to the hg19 reference genome, and variant calling with a GATK-based workflow, followed by functional annotation using ANNOVAR (version:2024Jun02). Variant interpretation was curated against thyroid cancer–related resources. All testing was conducted in the hospital’s molecular diagnostics laboratory under established quality assurance procedures

### 2.4. Surgical Procedures

Thyroid surgery was performed by experienced thyroid surgeons using either an open or an endoscopic approach, according to surgeon expertise and patient preference. The extent of surgery followed American Thyroid Association recommendations and our institutional protocol. In this cohort, the typical procedure consisted of unilateral lobectomy with isthmusectomy combined with routine central compartment lymph node dissection (CND, level VI). Lateral neck dissection (LND) was not performed routinely and was undertaken selectively when preoperative ultrasound suggested lateral nodal involvement and/or when intraoperative assessment indicated lateral disease.

### 2.5. Model Development and Validation

To predict the risk of cervical lymph node metastasis (CLNM) in PTC patients, we compared six representative algorithms spanning complementary families suitable for tabular clinical data—linear (logistic regression), distance-based (k-nearest neighbors), margin-based non-linear (RBF-SVM), tree-based ensembles (XGBoost, LightGBM), and a shallow neural network—to ensure diversity of inductive biases and capture both linear and non-linear effects. To optimize the predictive models, based on 10 rounds of 10-fold cross-validation and the default hyperparameter grid of the “caret” package, the final hyperparameters for each model were obtained on the optimal feature subset. Finally, the models were refit on the training set with the optimal feature subset and the final hyperparameters (based on 10 rounds of 10-fold internal cross-validation).

### 2.6. Statistical Analysis

Patients were randomly allocated 7:3 to training and validation sets; between-set differences were assessed using χ^2^ or Fisher’s exact tests for categorical variables, and non-normal data were summarized as median (IQR), while the *t*-test or the rank-sum test was used for continuous variables. An independent regression method was used to screen independent risk factors for cervical lymph node metastasis. Model performance was evaluated using receiver operating characteristic (ROC) curves and calibration curves, and decision curve analysis (DCA) was subsequently performed to determine the threshold of net benefit, including the area under the receiver operating characteristic curve (AUC), accuracy, precision, sensitivity, specificity, and F1 score. Final model selection was determined a priori by test-set performance to minimize overfitting, prioritizing F1 and then AUC. The model with the highest F1 score was considered the best-performing model; if F1 scores were identical, AUC values were compared. Calibration curves and DCA curves visualized the net benefit of the models under different thresholds. SHapley additive explanations (SHAP) values were used to determine the contribution of each feature to the final model. All analyses were performed in R (version 4.3.2; R Foundation for Statistical Computing). Reproducibility was ensured by setting a fixed random seed (1234) prior to data partitioning and model training.

## 3. Results

### 3.1. Baseline Clinical Information

Among 667 patients, females accounted for 75.7% and CLNM occurred in 391 (58.6%). The mean maximum tumor diameter was 1.08 cm (SD 0.73), and the mean BRAF V600E abundance was 21% (SD 12). Compared with the non-CLNM group, the CLNM group showed larger tumors (1.25 vs. 0.85 cm) and higher abundance (23% vs. 18%; both *p* < 0.001), and younger age (39 vs. 42 years, *p* < 0.001), and patients with microcalcifications, multifocality, and lymph node enlargement were more common in the CLNM group (all *p* < 0.05); meanwhile, the incidence of capsular invasion was also higher (*p* = 0.009). Details are shown in Table 1.

### 3.2. Independent Risk Factors

**Figure 2:**
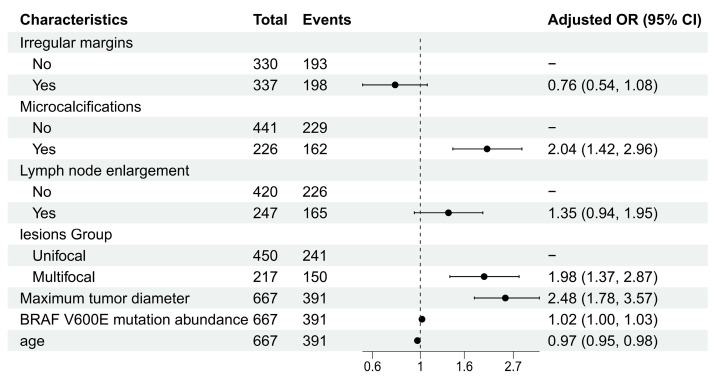
Forest plot of multivariable logistic regression analysis.

Using stepwise regression, we ultimately identified seven factors independently associated with CLNM: irregular margins, microcalcifications, lymph node enlargement, lesion group, maximum tumor diameter, BRAF V600E mutation abundance, and age. The specific odds ratios (ORs) are shown in Figure 2 (Table S1).

### 3.3. Dose–Response Relationship

**Figure 3:**
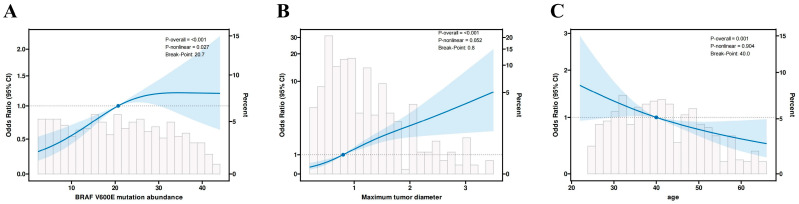
Restricted cubic spline (RCS) depiction of the association between (**A**) BRAF V600E mutation abundance, (**B**) maximum tumor diameter, and (**C**) age and the odds of cervical lymph node metastasis (CLNM). The solid line shows the estimated odds ratio; the shaded band denotes the 95% confidence interval. Light-gray histograms indicate the distribution of each predictor in the study cohort.

Based on the above stepwise regression results, we further used restricted cubic splines (RCS) to examine the dose–response relationships between the above continuous variables and CLNM risk, including BRAF V600E mutation abundance, maximum tumor diameter, and age. We used RCS for nonlinear testing to analyze the quantitative response relationships (Figure 3). The results showed a significant nonlinear relationship between BRAF V600E mutation abundance and cervical lymph node metastasis (overall *p* < 0.001, nonlinearity *p* = 0.027). Specifically, when mutation abundance increased from a low level to a moderate level (20.7%), the risk of cervical lymph node metastasis rose rapidly; when mutation abundance increased further, the risk began to flatten or decline. Maximum lesion diameter and age in PTC patients showed linear relationships with cervical lymph node metastasis (overall *p* < 0.05, nonlinearity *p* > 0.05), with risk thresholds of 0.8 (cm) and 40 (years), respectively.

### 3.4. Model Development and Performance Comparison

**Figure 4:**
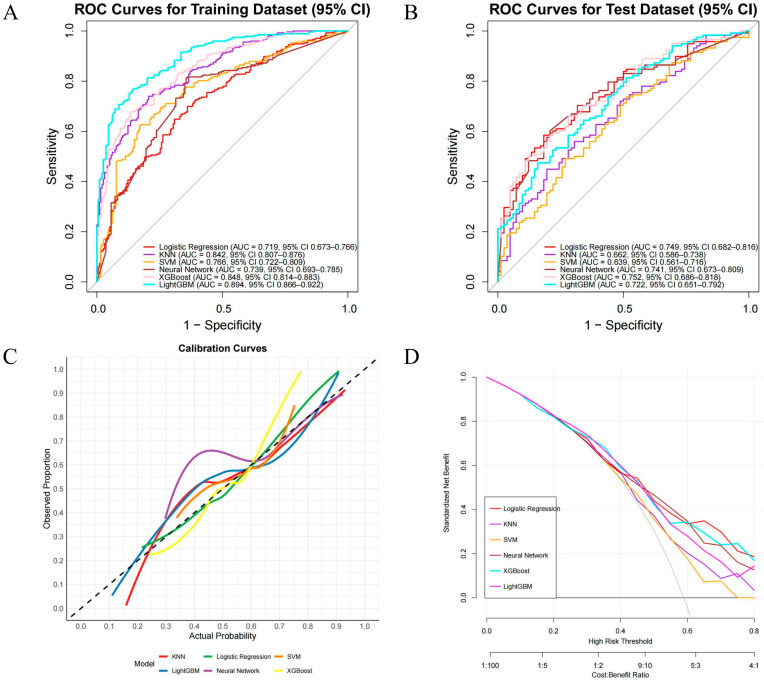
Performance of ML models for predicting CLNM in PTC. (**A**) ROC—training set; (**B**) ROC—test set; (**C**) calibration curves on the test set; the dashed 45° line indicates perfect calibration; (**D**) decision-curve analysis (DCA) across threshold probabilities 0.00–0.80, showing standardized net benefit.

After determining the key variables, we constructed six machine learning (ML) models for performance comparison, namely LR, KNN, SVM, XGB, LightGBM, and NN (Figure 4). ROC curves and AUCs were computed for the training and validation sets (Figure 4A,B); F1 scores (the harmonic mean of sensitivity and precision) were also calculated, and F1 scores were prioritized when comparing the discriminative performance of the six algorithms, followed by AUC values. In the training set, LightGBM performed best (AUC = 0.894, 95% CI 0.866–0.922), with sensitivity and specificity of 0.89 and 0.66, respectively; it was followed by XGBoost (AUC = 0.848, 95% CI 0.814–0.883), with an F1 score of 0.84. In the test set, XGBoost performed best (AUC = 0.752, 95% CI 0.686–0.818), with sensitivity and specificity of 0.78 and 0.49, respectively, and an F1 score of 0.73 (Table S2). Therefore, XGBoost (version 1.7.11.1), which performed best in the test set, was ultimately chosen as the optimal predictive model for subsequent clinical risk stratification and decision support. In addition, we used calibration curves (Figure 4C) to evaluate the accuracy of the predicted probabilities, and decision curve analysis (Figure 4D) showed that XGBoost had higher net benefit across clinically relevant thresholds.

### 3.5. Model Interpretation

**Figure 5:**
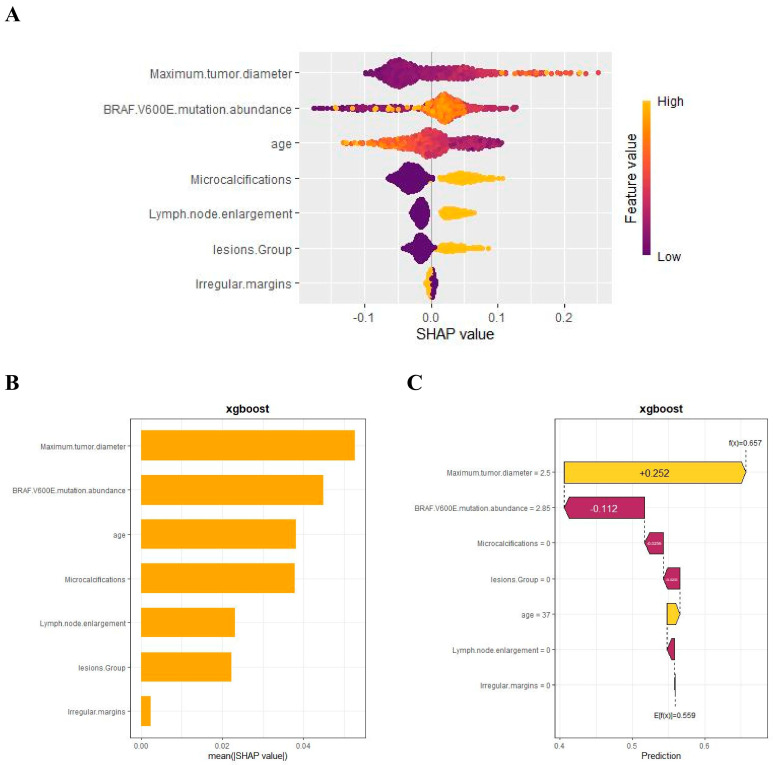

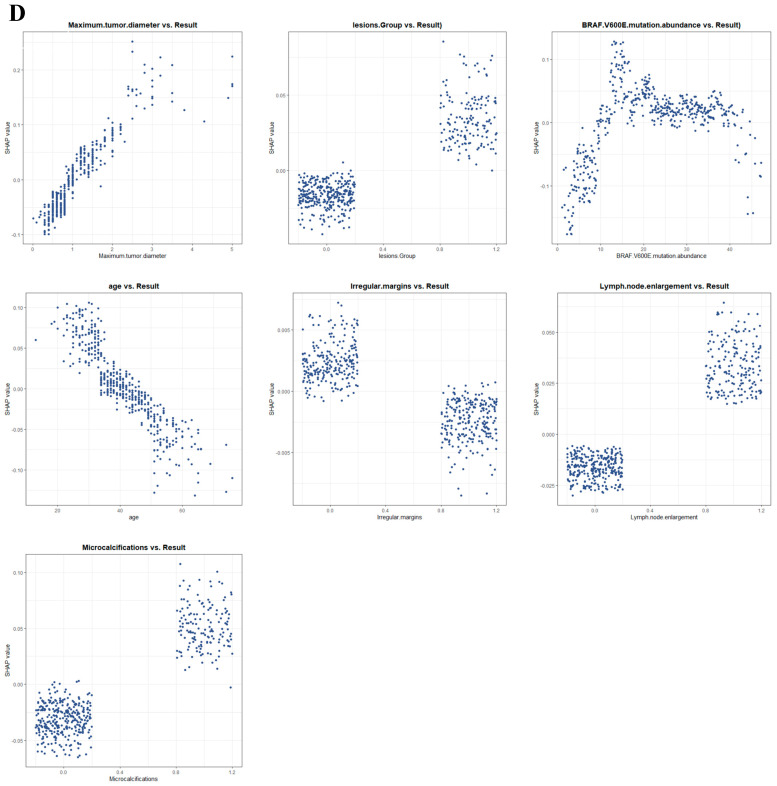
SHAP-based interpretation of the final model at the global and local levels: (**A**) summary dot plot; (**B**) summary bar plot; (**C**) waterfall plot; (**D**) dependence plots.

We used SHAP to interpret the output of the final model by calculating the contribution of each variable to the prediction. The SHAP summary dot plot (Figure 5A) shows that larger tumor diameter, higher mutation abundance, younger age, and the presence of microcalcifications significantly increased the risk of CLNM in PTC. The SHAP summary bar chart (Figure 5B), which uses mean SHAP values to evaluate feature contributions and displays them in descending order, indicates that maximum lesion diameter, BRAF V600E mutation abundance, age, and microcalcifications are the four most important features in the predictive model. Irregular margins showed an independent association in the multivariable model but contributed less to the SHAP ranking (Figure 5B), likely due to partial collinearity with other sonographic primitives. SHAP dependence plots illustrate how individual features affect the model’s output. In Figure 5D, the actual values of these seven features are compared with the SHAP values; higher SHAP values indicate higher predicted risk of lymph node metastasis. For example, larger tumor diameter and younger age push the prediction toward cervical lymph node metastasis; the presence of microcalcifications, multifocal lesions, and lymph node enlargement also promotes a positive prediction for CLNM. For BRAF V600E mutation abundance, when the abundance is <10%, most SHAP values are below zero, indicating lower risk; when the abundance is 10–20%, SHAP values increase rapidly; subsequently, as abundance continues to increase, SHAP values remain greater than zero, indicating a sustained and significant positive effect of high abundance on the risk of CLNM, with a certain plateau phase.

In addition, the SHAP waterfall plot (Figure 5C) shows the contribution of each feature to the model’s prediction of CLNM for a randomly selected PTC patient (Case No. 52). The specific values of each feature and their corresponding SHAP values indicate the positive and negative effects of that feature on the prediction. By accumulating SHAP values, the waterfall plot intuitively presents the formation process of the prediction for a specific patient, helping us to gain deeper insight into the model’s decision-making mechanism.

## 4. Discussion

This study, based on 667 patients with papillary thyroid carcinoma (PTC), integrated BRAF V600E mutation abundance with clinical data and developed multiple machine learning models to predict the risk of cervical lymph node metastasis (CLNM). The results showed that BRAF V600E mutation abundance was significantly higher in the CLNM group than in the non-metastatic group (median 23% vs. 17%, *p* < 0.001), suggesting a close association between higher mutation abundance and lymph node metastasis. This finding indicates that, beyond the binary “presence/absence” of BRAF V600E, its abundance—as a continuous variable—more effectively reflects tumor biology. Among the six models compared, XGBoost performed best, with an AUC of 0.848 in the training set and the highest F1 score of 0.73 and AUC of 0.75 in the test set, outperforming traditional logistic regression and other algorithms. In view of the combined performance on F1 and AUC, XGBoost was ultimately selected to build the predictive model. Notably, RCS analysis revealed a nonlinear dose–response relationship between BRAF V600E mutation abundance and CLNM risk: as abundance increased from a low level to approximately 20.7%, the risk of lymph node metastasis rose rapidly, whereas beyond this threshold the risk tended to plateau or even slightly decline. This “threshold effect” indicates that the driving effect of BRAF mutation on metastasis risk is most pronounced at intermediate abundance, whereas very high abundance may reflect different biological behavior. Mechanistically, the plateau at very high BRAF V600E abundance may reflect a ceiling (saturation) of MAPK/ERK pathway output with adaptive negative feedback once a dominant mutant clone is established, such that further increases in mutant-allele burden add little incremental invasiveness [33]. An alternative, not mutually exclusive interpretation is clonal sweep with reduced subclonal diversity; despite sustained MAPK activation, the loss of competing subclones may not further elevate the propensity for nodal dissemination [34]. Together, these hypotheses can be tested by multi-omic profiling (e.g., co-mutations such as TERT, copy-number burden, and immune contexture) and by external validation in prospective cohorts. By incorporating BRAF V600E mutation abundance as a continuous variable into the model and quantifying its nonlinear effect, this study achieved superior performance and provided new biological insights for risk assessment of CLNM in PTC compared with conventional approaches.

Prior studies on the association between BRAF V600E and PTC aggressiveness have yielded inconsistent conclusions: most reports link BRAF positivity to adverse outcomes such as lymph node metastasis, whereas some studies found no significant association, leaving the conclusions controversial [25,27,28,35,36]. This controversy may stem from viewing BRAF mutation solely as a binary variable (present or absent), without considering differences in intratumoral mutant clonal burden. The innovation of this study lies in introducing the concept of mutation abundance, using a continuous variable to reflect tumor heterogeneity and mutational burden, thereby enabling a more refined evaluation of the impact of BRAF mutation on metastasis [37]. Our results confirm that high-abundance BRAF V600E significantly increases the risk of CLNM and establishes an approximate risk threshold of 20.7%, indicating that BRAF V600E mutation abundance may need to accumulate to a certain level before disease occurrence is promoted [38,39]. This echoes prior evidence indicating that higher BRAF V600E mutation abundance is linked to more aggressive behavior [40]. However, Wang et al. considered the risk to increase linearly with abundance, without an obvious inflection point; in contrast, our RCS analysis revealed a significant nonlinear relationship, suggesting a sharp risk surge at intermediate abundance and a plateau at high abundance. In addition, Abdulhaleem et al. found in a study of PTC that tumors with aggressive pathological features had a mean BRAF V600E abundance as high as 25.8%, significantly higher than 10.3% in non-aggressive tumors, and that patients with lymph node metastasis had markedly higher mutation abundance (approximately 29% vs. 17%) [41]. Although they did not delineate the dose–response relationship of BRAF V600E mutation abundance, they proposed that BRAF mutation abundance could serve as an indicator for preoperative assessment of tumor aggressiveness and determination of surgical extent, which is consistent with our findings. Prior paired-sample studies report high concordance of BRAF V600E between preoperative FNA and matched surgical specimens (~90–98%), supporting the reliability of FNA-based molecular testing for our abundance-based risk modeling [42,43]. Collectively, these results indicate that the presence of BRAF V600E alone does not necessarily lead to disease, and that the degree of mutation must also be considered. Differences in dose–response patterns across studies may result from variations in sample size and analytical methods; with validation in larger samples, the “threshold effect” of BRAF mutation abundance is expected to be further confirmed. In addition, by incorporating BRAF abundance into a multivariable machine learning model, this study markedly improved predictive performance, providing a useful complement to previous models that relied solely on clinical imaging features [35,36,37,38,39,40,41,44].

Beyond BRAF mutation abundance, the other key predictors identified by the model are consistent with PTC biology. By definition, BRAF V600E abundance represents the proportion of mutant alleles among the total measured alleles, comprehensively reflecting tumor cell clonal architecture and tumor purity; an increase indicates dominance of the mutant clone, with more cells persistently in an activated MAPK/ERK pathway state, thereby elevating overall invasive potential. Multiple studies support this mechanism: Huang et al. found in PTC patients with intermediate-to-high recurrence risk that for each 1% increase in BRAF V600E mutation abundance, the risk of recurrence increased by approximately 8%, and proposed a threshold of 28.2% for recurrence risk stratification; Schumm et al., in JCEM, reported a significant association between high mutation abundance and adverse pathological outcomes and emphasized its clinical reportability; Tatar et al. also showed that tumors with high mutation abundance are closely associated with more aggressive behavior and higher risk of lymph node metastasis [40,45,46]. Consistent with these findings, we identified an abundance threshold of approximately 20.7% corresponding to a significant increase in CLNM risk, which constitutes a callback to the RCS conclusion in the first paragraph. In terms of interpretability, SHAP (SHapley Additive exPlanations, SHAP) bar plots showed that maximum tumor diameter, microcalcifications, multifocality, lymph node enlargement, and younger age were the most important contributors; their directions are concordant with findings from Liu et al., who reported in a multicenter prospective study that maximum diameter > 10 mm was a predictor of CLNM with OR = 1.81 (*p* < 0.001), and from Han et al., who reported that microcalcifications, age < 45 years, and multifocality increase the risk of lymphatic metastasis [35,47]. These points outline the significance of our study and its relation to prior work. Thus, the two tracks of performance and biology converge here, indicating that this continuous measure has practical stratification value.

Nevertheless, we acknowledge several limitations and uncertainties. First, this was a single-center retrospective study without an external validation set; its generalizability needs to be examined in prospective, multicenter cohorts. Because quantitative BRAF V600E mutation–abundance testing is embedded in our preoperative pathway, entry-into-testing selection bias and unmeasured confounding cannot be fully excluded. We plan cell-based BRAF V600E mutation abundance assays and a multicenter registry to validate/recalibrate the model and reassess the ~20% threshold/plateau. Second, the current model considered only the BRAF V600E mutation and did not integrate other potentially important molecular features, such as TERT mutations, RAS mutations, RET/PTC fusions, and immune markers; future work should expand toward multi-gene and multimodal model construction. Third, long-term follow-up has not yet been completed, and the model’s value in predicting postoperative recurrence, enabling dynamic monitoring, and assessing treatment response remains to be verified. Finally, although SHAP provides strong model interpretability, the causal mechanisms for some features require further mechanistic studies. The apparent plateau beyond ~20.7% is hypothesis-generating—possibly reflecting pathway saturation or immune surveillance—but requires external validation and mechanistic confirmation. We will investigate this in subsequent basic experiments, and jointly incorporate mutation abundance, radiomics, and immune transcriptomic features to build a multidimensional predictive framework integrating genomic data, artificial intelligence, and radiomics, with a prospective design to evaluate its potential for clinical decision support; this also echoes the earlier note that the “threshold effect” requires external validation.

This interpretable CLNM risk prediction model shows promise for clinical translation. Although the RCS curve delineates clinically intuitive risk bands for BRAF V600E mutation abundance—for example, <10% (low), 10–20.7% (intermediate), and ≥20.7% (high)—we regard these bands as aids for communication and stratification rather than prescriptive cut points. In practice, a low predicted risk (<10%) generally supports conservative cervical nodal management with routine surveillance; a high predicted risk (≥20.7%) would prompt consideration of formal preoperative cervical nodal mapping and multidisciplinary team (MDT) discussion, more often favoring operative management; and an intermediate estimate (10–20.7%) warrants individualized appraisal that integrates tumor size, ultrasound features (e.g., microcalcifications, suspicious nodes), cytology/Bethesda category, comorbidities, and patient preferences. These bands should be locally recalibrated using decision-curve analysis alongside local service constraints. Importantly, the model is an aid—not a directive—and the proposed bands remain exploratory pending external validation and mechanistic studies. In preoperative decision-making, the model helps identify high-risk individuals and thereby assists in formulating individualized surgical plans; given that the incidence of CLNM in PTC can be as high as 30–80%, and that preoperative imaging has limited sensitivity for detecting micrometastases [48], performing a second operation after postoperative detection increases patient burden and the risk of complications [49], making earlier risk stratification of practical significance. From the perspective of precision medicine, quantitative BRAF V600E abundance provides an individual with a molecular risk indicator, aligning with the concept of stratification by molecular features; moreover, the interpretability of the model (including SHAP plots) facilitates physician–patient communication by visually presenting the main factors driving individual risk, which helps achieve an actionable management plan. Overall, we prefer to regard this as an actionable starting point to be integrated in parallel with clinical experience and resource constraints, rather than as a predetermined single endpoint.

## 5. Conclusions

In summary, our study confirms a nonlinear dose–response relationship between BRAF V600E mutation abundance and the risk of lymph node metastasis in PTC, with an abundance threshold of approximately 20.7% being associated with a markedly increased metastasis risk; a machine learning model that combines mutation abundance with clinical features can effectively predict lymph node metastasis risk, with good interpretability and potential clinical applicability.

## Figures and Tables

**Table 1:** Baseline characteristics of 667 patients with papillary thyroid carcinoma.

Characteristic	CLNM			*p*-Value
	Overall N = 667	No N = 276	Yes N = 391	
Age, Mean ± SD	41 ± 11	43 ± 11	40 ± 11	<0.001 ^1^
Maximum tumor diameter				
Mean ± SD	1.08 ± 0.73	0.85 ± 0.50	1.25 ± 0.82	<0.001 ^1^
BRAF V600E mutation abundance				
Mean ± SD	21 ± 12	18 ± 12	23 ± 11	<0.001 ^1^
sex, n (%)				0.027 ^2^
Male	162 (24.3%)	55 (19.9%)	107 (27.4%)	
Female	505 (75.7%)	221 (80.1%)	284 (72.6%)	
Irregular margins, n (%)				0.944 ^2^
No	330 (49.5%)	137 (49.6%)	193 (49.4%)	
Yes	337 (50.5%)	139 (50.4%)	198 (50.6%)	
Hypoechoic, n (%)				0.907 ^2^
No	93 (13.9%)	39 (14.1%)	54 (13.8%)	
Yes	574 (86.1%)	237 (85.9%)	337 (86.2%)	
Microcalcifications, n (%)				<0.001 ^2^
No	441 (66.1%)	212 (76.8%)	229 (58.6%)	
Yes	226 (33.9%)	64 (23.2%)	162 (41.4%)	
Tumor aspect ratio, n (%)				0.125 ^2^
<1	345 (51.7%)	133 (48.2%)	212 (54.2%)	
>1	322 (48.3%)	143 (51.8%)	179 (45.8%)	
CDFI, n (%)				0.015 ^2^
Poor blood supply	604 (90.6%)	259 (93.8%)	345 (88.2%)	
Rich blood supply	63 (9.4%)	17 (6.2%)	46 (11.8%)	
Lymph node enlargement, n (%)				0.001 ^2^
No	420 (63.0%)	194 (70.3%)	226 (57.8%)	
Yes	247 (37.0%)	82 (29.7%)	165 (42.2%)	
Ultrasound classification, n (%)				0.021 ^3^
2	1 (0.1%)	1 (0.4%)	0 (0.0%)	
3	15 (2.2%)	7 (2.5%)	8 (2.0%)	
4	146 (21.9%)	74 (26.8%)	72 (18.4%)	
5	505 (75.7%)	194 (70.3%)	311 (79.5%)	
Lesion Group, n (%)				<0.001 ^2^
Unifocal	450 (67.5%)	209 (75.7%)	241 (61.6%)	
Multifocal	217 (32.5%)	67 (24.3%)	150 (38.4%)	
Capsule invasion, n (%)				0.009 ^2^
No	148 (22.2%)	75 (27.2%)	73 (18.7%)	
Yes	519 (77.8%)	201 (72.8%)	318 (81.3%)	

^1^ Welch Two-Sample *t*-Test. ^2^ Pearson’s Chi-Squared Test. ^3^ Fisher’s Exact Test.

## Data Availability

The analysis code and a synthetic demonstration dataset are openly available in the Open Science Framework (OSF) repository at: https://osf.io/n37ch/?view_only=32723588ee2f4c0aa7b5a5854003b4b3 (accessed on 17 September 2025). The demo dataset reproduces the analysis workflow and expected file structure; its outputs verify functionality and do not match the manuscript’s numerical results. The full de-identified patient-level dataset underlying the findings cannot be made publicly available due to institutional ethics and privacy restrictions. Access for editors and peer reviewers can be provided under controlled conditions in accordance with the approved protocol (IRB: SYSKY-2024-169-01). Subject to ethics approval and a data use agreement, qualified investigators may obtain the dataset upon reasonable request to the corresponding author.

## Supplementary Materials

The following supporting information can be downloaded at https://www.mdpi.com/article/10.3390/cancers17213562/s1, Supplementary Table S1 (DOCX): Independent risk factors for CLNM in PTC identified by multivariable logistic regression (odds ratio [OR] with 95% confidence intervals); Supplementary Table S2 (DOCX): Performance of six machine-learning models on the training and test sets (AUC, F1 score, accuracy, sensitivity, specificity, PPV, NPV).

## Abbreviations

The following abbreviations are used in this manuscript:

AUC	Area under the receiver operating characteristic curve
CDFI	Color Doppler flow imaging
CI	Confidence interval
CLNM	Cervical lymph node metastasis
DCA	Decision curve analysis
F1	F1 score
FNA	Fine-needle aspiration
KNN	k-nearest neighbors
LightGBM	Light Gradient Boosting Machine
LR	Logistic regression
MAPK	Mitogen-activated protein kinase
ML	Machine learning
NN	Neural network
OR	Odds ratio
PTC	Papillary thyroid carcinoma
RAS	RAS proto-oncogene family
RET/PTC	RET proto-oncogene/Papillary Thyroid Carcinoma fusion
RCS	Restricted cubic splines
ROC	Receiver operating characteristic
SHAP	SHapley Additive exPlanations
SVM	Support vector machine
TI-RADS	Thyroid Imaging Reporting and Data System
XGB	Extreme Gradient Boosting (XGBoost)
