# Supplementary material for: Dose–Response Relationship Between BRAF V600E Abundance and Cervical Lymph Node Metastasis in Papillary Thyroid Cancer
Source: Cancers (Basel). 2025 Nov 3;17(21):3562. doi: 10.3390/cancers17213562 (PMC12607434; doi:10.3390/cancers17213562)
Supplement: Supplementary file 1 [file cancers-17-03562-s001.zip › cancers-3918286-supplementary/tabel S1.pdf]

Supplementary Table S1. Independent risk factors for cervical lymph node metastasis in papillary thyroid carcinoma identified by multivariable logistic regression (OR, 95% Table S1

Multivariate Logistic Regression

Characteristic	N	Event N	OR	95% CI	p-value
**Irregular margins**					
No	330	193	—	—	
Yes	337	198	0.76	0.54, 1.08	0.126
**Microcalcifications**					
No	441	229	—	—	
Yes	226	162	2.04	1.42, 2.96	<0.001
**Lymph node enlargement**					
No	420	226	—	—	
Yes	247	165	1.35	0.94, 1.95	0.107
**lesions Group**					
Unifocal	450	241	—	—	
Multifocal	217	150	1.98	1.37, 2.87	<0.001
**Maximum tumor diameter**	667	391	2.48	1.78, 3.57	<0.001
**BRAF V600E mutation abundance**	667	391	1.02	1.00, 1.03	0.029
**age**	667	391	0.97	0.95, 0.98	<0.001

Abbreviations: CI = Confidence Interval, OR = Odds Ratio
