# Supplementary material for: Dose–Response Relationship Between BRAF V600E Abundance and Cervical Lymph Node Metastasis in Papillary Thyroid Cancer
Source: Cancers (Basel). 2025 Nov 3;17(21):3562. doi: 10.3390/cancers17213562 (PMC12607434; doi:10.3390/cancers17213562)
Supplement: Supplementary file 1 [file cancers-17-03562-s001.zip › cancers-3918286-supplementary/table S2.pdf]

Supplementary Table S2. Performance of six machine-learning models on training and validation sets: AUROC (95% CI), accuracy, sensitivity, specificity, F1-score, PPV, and NPV for preoperative prediction of cervical lymph node metastasis in papillary thyroid carcinoma.

**training**	**AUC**	**95% CI**	**Accuracy**	**Sensitivity**	**Specificity**	**F1**	**PPV**	**NPV**
Logistic	0.719	0.673 – 0.766	0.6638	0.7656	0.5206	0.73	0.6921	0.6121
KNN	0.842	0.807 – 0.876	0.7473	0.8498	0.6031	0.8	0.7508	0.7405
Xgboost	0.848	0.814 – 0.883	0.758	0.8681	0.6031	0.81	0.7548	0.7647
LightGBM	0.894	0.866 – 0.922	0.7987	0.8938	0.6649	0.84	0.7896	0.8165
SVM	0.766	0.722 – 0.809	0.6959	0.8205	0.5206	0.76	0.7066	0.6733
NNET	0.739	0.693 – 0.785	0.7409	0.8132	0.6392	0.79	0.7603	0.7086
**testing**	**AUC**	**95% CI**	**Accuracy**	**Sensitivity**	**Specificity**	**F1**	**PPV**	**NPV**
Logistic	0.749	0.682 – 0.816	0.67	0.7542	0.5488	0.73	0.7063	0.6081
KNN	0.662	0.586 – 0.738	0.625	0.6949	0.5244	0.69	0.6777	0.5443
Xgboost	0.752	0.686 – 0.818	0.66	0.7797	0.4878	0.73	0.6866	0.6061
LightGBM	0.722	0.651 – 0.792	0.66	0.7458	0.5366	0.72	0.6984	0.5946
SVM	0.639	0.561 – 0.716	0.625	0.7458	0.4512	0.7	0.6617	0.5522
NNET	0.741	0.673 – 0.809	0.685	0.7288	0.622	0.73	0.735	0.6145
